# ^1^H-NMR dataset for hydroxycoumarins –Aesculetin, 4-Methylumbelliferone, and umbelliferone

**DOI:** 10.1016/j.dib.2016.05.048

**Published:** 2016-05-30

**Authors:** Rui Galhano dos Santos, João Carlos Moura Bordado, Maria Margarida Mateus

**Affiliations:** CERENA, Departamento de Engenharia Química e Biológica, Torre Sul, Instituto Superior Técnico, Av. Rovisco Pais, 1049-001 Lisboa, Portugal

**Keywords:** Coumarins, NMR, Acetone

## Abstract

Herein, the integrated raw data regarding the ^1^H-NMR, experiments of Aesculetin, 4-Methylumbelliferone, and umbelliferone, in Acetone-d^6^ at 25 °C, are presented for further analysis and comparison purposes, for whom may be interested.

**Specifications Table**

**Table:** 

Subject area	*Chemistry*
More specific subject area	*Structural characterization*
Type of data	*Figures, table*
How data was acquired	*The data was acquired on a Bruker Avance 400 spectrometer operating at 400 MHz*
Data format	*Raw*
Experimental factors	*Sample solutions were prepared with deuterated*
	*Acetone (Acetone-d*^*6*^*). Residual acetone peak: 2.05*
Experimental features	*Detection temperature was set at 25 °C. Samples were scanned 16 times.*
Data source location	*Lisbon, Portugal, GPS: 38° 44׳ 10.31׳׳N; 9° 08׳ 19.66׳׳W*
Data accessibility	*Data is provided in the article*

**Value of the data**

• Useful to be used as reference data for chemical shifts for other related compounds.
• Comparison between coumarins with different substitution patterns.
• Helpful in the assignment of signals of molecules containing coumarin backbone residues.

## 1 Data

The data henceforth described refers to the ^1^H-NMR experiments of three coumarins, Aesculetin, 4-Methylumbelliferone, and umbelliferone, in deuterated acetone.

**Fig. 1:**
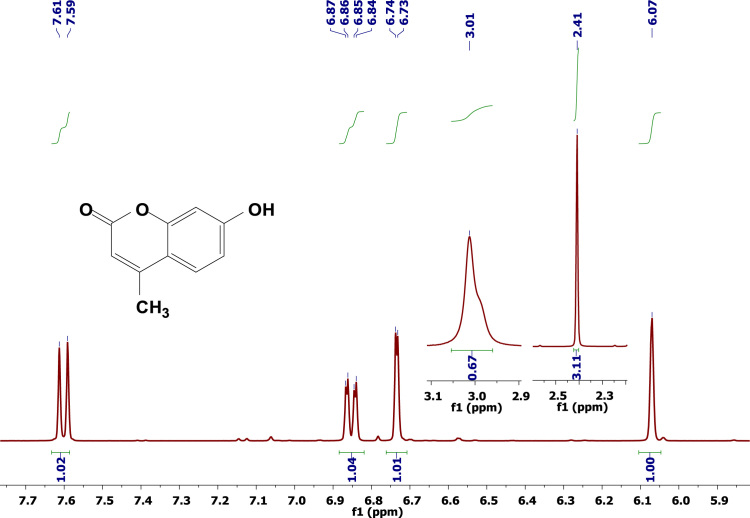
^1^H-NMR spectrum of 4-Methylumbelliferone (acetone-d^6^, 298 K, 400 MHz).

**Fig. 2:**
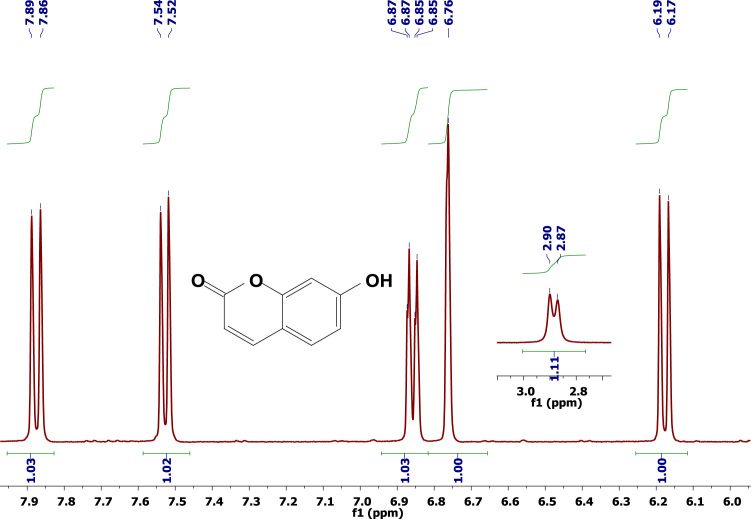
^1^H-NMR spectrum of umbelliferone (acetone-d^6^, 298 K, 400 MHz).

**Fig. 3:**
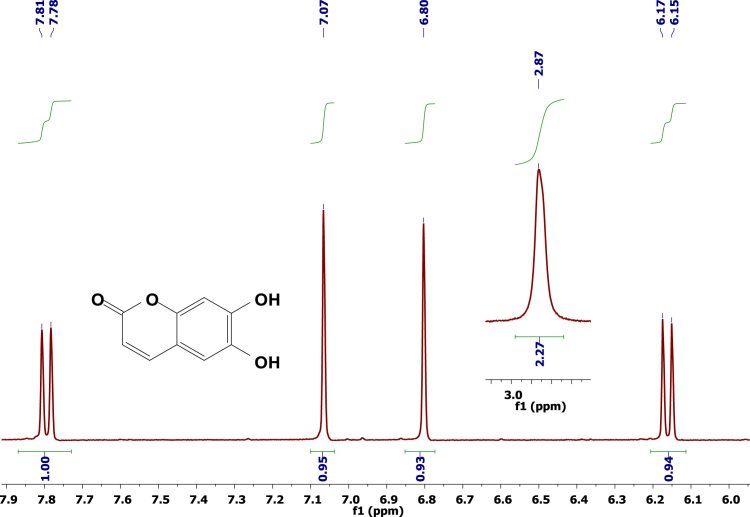
^1^H-NMR spectrum of Aesculetin (acetone-d^6^, 298 K, 400 MHz).

The data disclosed regards the ^1^H-NMR experiments conducted with 4-Methylumbelliferone (Fig. 1), umbelliferone (fig. 2), and Aesculetin (Fig. 3), in deuterated acetone. This data may be helpful for those who intend to compare this data with other from molecules containing the same or related coumarins scaffolds. Table 1 lists all the peaks and their respective intensities.

## 2 Experimental design, materials, and methods

The coumarins used were chemical grade and purchased from Sigma-Aldrich.

The compounds were subjected to ^1^H-NMR measurements. The experiments were performed on a Bruker Avance 400 liquid NMR spectrometer, operating at 400 MHz. Detection temperature was set at 25 ^o^C. The samples were loaded in a 5 mm NMR tube. The solvent peak was calibrated according to Gottlieb et al. [1].

## Figures and Tables

**Table 1:** Chemicals shifts for 4-methylumbelliferone, umbelliferone, and Aesculetin [1].

**Compound**					
**4-Methylumbelliferone**		**Umbelliferone**		**Aesculetin**	
**ppm**	**Intensity**	**ppm**	**Intensity**	**ppm**	**Intensity**
2.41	2129.6	2.87	171.2	2.87	134.2
3.01	116.5	2.9	194.3	6.15	246.5
6.07	607.4	6.17	501.6	6.17	255.4
6.73	518.7	6.19	515.2	6.8	456
6.74	535.3	6.76	662.3	7.07	484.5
6.84	291.3	6.85	379.1	7.78	237.5
6.85	251.3	6.87	274.9	7.81	233.3
6.86	308.3	7.52	511.3	–	–
6.87	268.4	7.54	479.5	–	–
7.59	489.4	7.86	484.7	–	–
7.61	461.7	7.89	471.7	–	–
9.35	70.5	–	–	–	–
